# Assessing the Effect of Quantitative and Qualitative Predictors on Gastric Cancer Individuals Survival Using Hierarchical Artificial Neural Network Models

**DOI:** 10.5812/ircmj.4122

**Published:** 2013-01-05

**Authors:** Zohreh Amiri, Kazem Mohammad, Mahmood Mahmoudi, Mahbubeh Parsaeian, Hojjat Zeraati

**Affiliations:** 1 Department Of Basic Sciences, National Nutrition and Food Technology Research Institute, Faculty of Nutrition Sciences and Food Technology, Shahid Beheshti University of Medical Sciences, Tehran, IR Iran; 2 Department of Epidemiology and Biostatistics, School of Public Health, Tehran University of Medical Sciences, Tehran, IR Iran

**Keywords:** Survival, Life Expectancy, Proportional Hazards Model, Neural Networks

## Abstract

**Background:**

There are numerous unanswered questions in the application of artificial neural network models for analysis of survival data. In most studies, independent variables have been studied as qualitative dichotomous variables, and results of using discrete and continuous quantitative, ordinal, or multinomial categorical predictive variables in these models are not well understood in comparison to conventional models.

**Objectives:**

This study was designed and conducted to examine the application of these models in order to determine the survival of gastric cancer patients, in comparison to the Cox proportional hazards model.

**Patients and Methods:**

We studied the postoperative survival of 330 gastric cancer patients who suffered surgery at a surgical unit of the Iran Cancer Institute over a five-year period. Covariates of age, gender, history of substance abuse, cancer site, type of pathology, presence of metastasis, stage, and number of complementary treatments were entered in the models, and survival probabilities were calculated at 6, 12, 18, 24, 36, 48, and 60 months using the Cox proportional hazards and neural network models. We estimated coefficients of the Cox model and the weights in the neural network (with 3, 5, and 7 nodes in the hidden layer) in the training group, and used them to derive predictions in the study group. Predictions with these two methods were compared with those of the Kaplan-Meier product limit estimator as the gold standard. Comparisons were performed with the Friedman and Kruskal-Wallis tests.

**Results:**

Survival probabilities at different times were determined using the Cox proportional hazards and a neural network with three nodes in the hidden layer; the ratios of standard errors with these two methods to the Kaplan-Meier method were 1.1593 and 1.0071, respectively, revealed a significant difference between Cox and Kaplan-Meier (P < 0.05) and no significant difference between Cox and the neural network, and the neural network and the standard (Kaplan-Meier), as well as better accuracy for the neural network (with 3 nodes in the hidden layer). Probabilities of survival were calculated using three neural network models with 3, 5, and 7 nodes in the hidden layer, and it has been observed that none of the predictions was significantly different from results with the Kaplan-Meier method and they appeared more comparable towards the last months (fifth year). However, we observed better accuracy using the neural network with 5 nodes in the hidden layer. Using the Cox proportional hazards and a neural network with 3 nodes in the hidden layer, we found enhanced accuracy with the neural network model.

**Conclusions:**

Neural networks can provide more accurate predictions for survival probabilities compared to the Cox proportional hazards mode, especially now that advances in computer sciences have eliminated limitations associated with complex computations. It is not recommended in order to adding too many hidden layer nodes because sample size related effects can reduce the accuracy. We recommend increasing the number of nodes to a point that increased accuracy continues (decrease in mean standard error), however increasing nodes should cease when a change in this trend is observed.

## 1. Background 

Human reasoning is a quest that has always been pursued by investigators, and many efforts have been made to design models that represent such processes. During the mid-decade of the twentieth century, advances in computers provided a foundation for more accurate assessments in this field; more information was collected regarding the functioning of the human nervous system, and thus, developing models was enabled. Mathematical modeling of the nervous system was expanded in studies by McLelland and Rummelhart during 1982 to 1987. Modeling neural networks (NN) is relatively new endeavor and their applications have been explored and discussed in recent years. Traditional statistical methods and NN models may emerge similar; however, the major difference between them is that traditional methods tend to focus on finding solutions to linear equations while NNs are mostly focused on the resolution non-linear solutions. Artificial NN can be considered as mathematical algorithms that make essential reasoning based on the limited information available in its primary units (1). In biological models, neurons are known as a primary processing element (PE). Each neuron processes an input and generates an output. Every NN receives a number of inputs and generates one or more outputs. The output of a neuron is dichotomous, however it can also be modeled as a continuous variable. The relationship between the input and output of a given neuron can be expressed using mathematical functions which explain the behavior of the neuron. The strength of the connection between neurons may vary in these models. Although the result of a given function with a fix input is known and fixed, the result of the relation between two neurons does not remain constant over time. Consequently, the function of a network of neurons and their relations are affected by these varying connections and the system is constantly changing and learning. The connections between neurons determine the behavior of the network and its temporal changes. PEs are usually organized within layers. In these models, there are frequently three layers; an input layer comprised of independent variables, an output layer related to dependent variables, and one or more middle layers known as the hidden units. Every PE in each layer is connected to all the PEs in the next layers. The main task in each NN model is to find model coefficients which can convert input to output in the middle layer(s) with minimum errors. Usually the weighted sum of the input plus a constant value (bias) in the middle layer is influenced by a fixed function (e.g. logistic). Weights are defined through minimizing the function of error by minimizing the sum of squares, or minus of Log Likelihood. The most common application for NN models in health and medical studies is in medical diagnosis, and few reports are exist on how these models are applied in medical studies. Particular studies concern outcomes of cardio-pulmonary resuscitation (2), success rates of drug detoxification programs (3), tumor progression in cancer research and the level of failure in liver transplant. The diagnosis of myocardial infarction has been studied by using the serum enzyme levels in a NN where a 100% sensitivity and 8% false positivity was observed (4, 5). The next group used not only enzyme levels but also electrocardiograms, and achieved more accurate predictions (6). The diagnosis of myocardial infarction has been studied by using the serum enzyme levels in a NN where a 100% sensitivity and 8% false positivity was observed (7). The next group used not only enzyme levels but also electrocardiograms, and achieved more accurate predictions (8). In terms of NN architecture, the most conventional model is one known as the multilayer perception (MLP) which includes a layer of input variables, and output layer, and one or more layers of hidden units. The application of such models has been very limited in survival studies. In 1992, Ravdin et al. were the first to practice these models in studying survival in breast cancer patients, and they demonstrated that these types of models can generate relatively more accurate results compared to traditional methods. In their studies, time was entered as a predictive variable, and for every patient, the number of time intervals the patient lived was considered a variable (9). In 1996, Ohno-Machado published a report concerning the application of a multiple neural network for processing survival data. In her study, data pertaining to the same given time and event, including censored data, were fit in the same NN, and the output of the network was studied in the more general model (10). In a report entitled "Neural networks as statistical methods in survival analysis", Ripley and Ripley discuss the application of NN in comparison to traditional methods using data from patients who suffered from breast cancer. Their main objective was to substitute a linear function with a neural network. In their opinion, NNs are powerful, and driving them well can be as difficult as driving a powerful car, therefore using a less complicated tool is more suitable to avoid confusion. They suggested that over fitting was the main challenge with NNs, and based on the sensitivity and specificity of the model, they concluded that there was insufficient evidence to claim NNs superior to other models (11). In "Non-linear survival analysis using neural networks" Ripley et al. report the application of NNs in the analysis of data from 1335 patients with breast cancer where survival time until the first relapse was studied as the dependent variable and 11 demographic, diagnostic, and treatment related variables were the independent variables. Missing independent variables were estimated through multiple linear regressions, and analyses were done using data from 680 patients who had no missing data. Variables were coded in a binary format, and analyses were based on multi-layer perception models which were free of the assumptions used in all regression models. The authors describe their use of seven different NNs and their performance in predicting the time to relapse in patients with breast cancer. In the first model, time to relapse was split into two intervals. They also had two models with time split into five intervals, and four models in which time to relapse was considered continuous (log logistic, proportional hazard, log normal, and an extension of the proportional hazards model). The authors concluded that utilizing the models was not very beneficial (12). In a different study, the authors of the present report used the same data and found no significant difference between NN and the traditional method by fitting dichotomous variables only (13).

In Iran, life expectancy of patients who suffer from gastric cancer has been investigated in various studies including one based on the data bank of the present project where the 5-year survival rate was 23.6% and the median life expectancy was 19.90 months. In these studies, investigators used the Cox proportional hazards model and found that age, presence of metastasis, and cancer stage may affect patient survival (14). As discussed above, Kaplan-Meier and Cox proportional hazards models are conventional models which can be easily applied using computer software; however, they require certain assumptions that are usually overlooked. Example challenges include assumptions for simplifying models (e.g. assumptions regarding the linearity of correlations and models), ignoring the interaction between independent variables, doubt about the proportionality assumption, uncertain distributions, as well as errors related to curve fitting (15, 16). NNs have been used in classification and failure prediction in the past 20 years; numerous applications have been found for NNs in classification, but their role in prediction analysis has received less attention (16, 17).

## 2. Objectives 

The present study aims to find out whether NNs are superior to Cox proportional hazard model and Kaplan-Meier when quantitative, ordinal or multinomial variables were included in the model.

## 3. Patients and Methods

In this study 330 patients with gastric cancer who had history of surgery at the Iran Cancer Institute over a 5-year period were enrolled and their postoperative life expectancy was determined. Those who were alive at the end of the study, and individuals who had missing data from the time they were lost to follow up, were excluded. During the study period, 239 patients died; the causes of which were not disease-related (in 13 cases) were considered as right censored from the date of death. Firstly, we randomly divided cases into two groups of 165 patients each to examine models and assess reproducibility; the training group and the study group. To ensure group similarity and random allocation, the chi-square test and multiple logistic regression analysis were used. Subgroups were compared using the log-rank test. Independent variables of age (continuous quantitative), gender (dichotomous: woman = 0, man = 1), history of substance abuse (dichotomous: no = 0, yes = 1), cancer site (trinomial: anterior = 2, cardia = 1, other = 0), type of pathology (dichotomous: adenocarcinoma = 1, other = 0), presence of metastasis (dichotomous: no = 0, yes = 1), T-stage (ordinal: 1-4), N-stage (ordinal: 0-3), M-stage (ordinal: 0-1), number of complementary treatments (discrete quantitative), and computed probabilities of survival by time were used. Subsequently, we calculated probabilities of survival past 6, 12, 18, 24, 36, 48, and 60 months using the Cox proportional hazards model and a NN. For this purpose, estimates and coefficients of the Cox model and the weights of the NN model (with 3, 5, and 7 nodes in the hidden layer) were determined in the training group and used to derive predictions in the study group. Predictions with these two methods were compared with those of the Kaplan-Meier product limit estimator as the gold standard. Comparisons were made with the Friedman and Kruskal-Wallis tests. Staging was done using the 6th edition of the TNM system. For statistical analyses, SPSS version 11, Matlab version 7.2, Statistica version 6.0, and S_PLUS 2000 were used and the level of significance was considered 0.05.

## 4. Results

**Figure 1.**
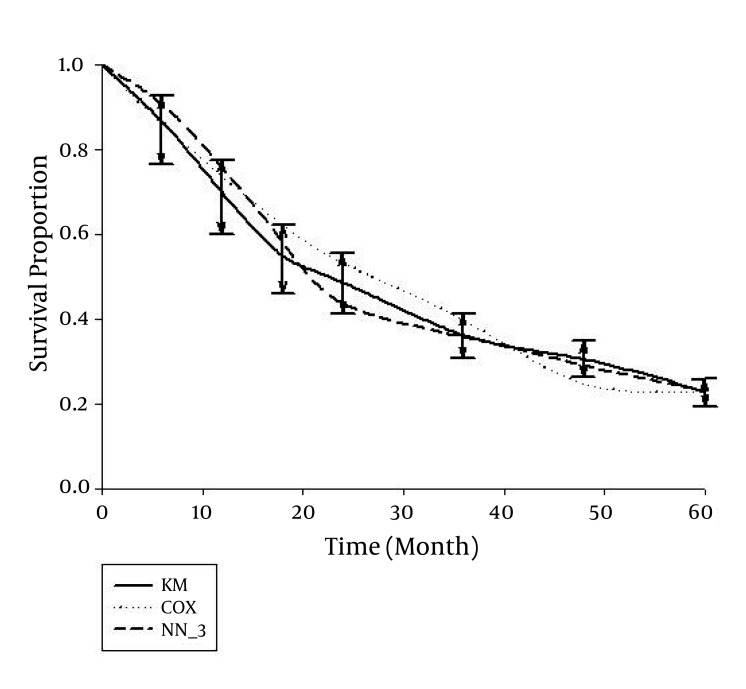
Survival probabilities in the training group predicted using Cox and neural network methods (with a 3-node hidden layer) (NN_3) compared to the Kaplan Meier (KM) method (95% limits of agreement)

**Figure 2.**
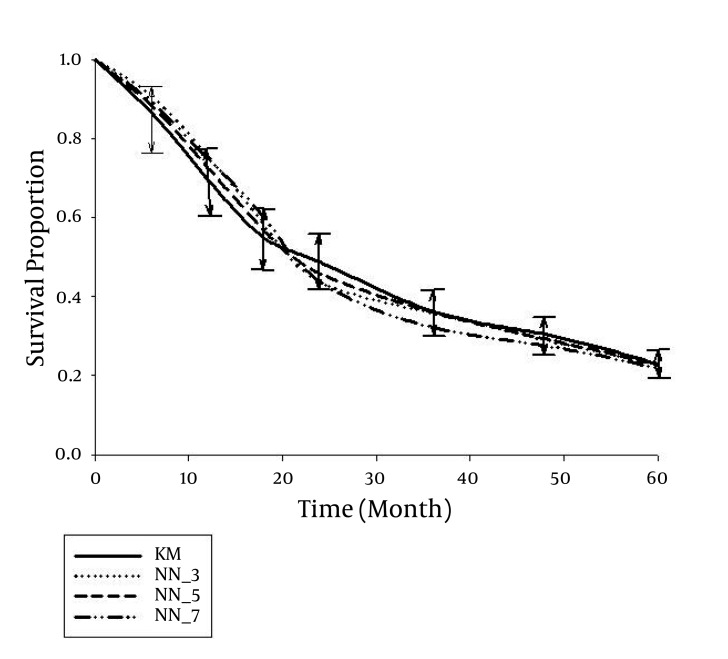
Survival probabilities in the study group predicted using three neural network based (NN) methods (with 3-, 5-, and 7-node hidden layers) compared to the Kaplan Meier (KM) method (95% limits of agreement)

The median age of the individuals was 68 years (range, 32 to 96 years) whereas 69.1% of them were male. The type of pathology was adenocarcinoma in 85.2%; other types included squamous cell carcinoma, small cell carcinoma, carcinoid carcinoma, sarcoma, stromal tumor, malignant lymphoma, and spindle cell tumor. Metastases were present in 192 cases (58.2%); the stage was IA in 3.0%, IB in 3.6%, II in 18.2%, IIIA in 13.0%, IIIB in 3.3%, and the disease stage in the remaining 58.8% was IV. In all stage IV cases, there was either N3 or T4, or there was T3 and M1. No complementary treatment was administered in 20.3% of patients, while 26.1% had received other treatments three times. The first and 5th-year survival rates were 66.7% and 23.6%, respectively, and the median life expectancy was 19.9 months (13, 14). In the first step, we randomly divided cases into two groups of 165 patients each. We used the chi-square test and the backward multiple logistic regression analysis (with significance levels of the Wald statistics between 0.14 and 0.77) to ensure there was no significant difference between the distributions of independent variables in the two groups (Table 1). We also applied the log-rank test to ensure that life expectancy in the two groups (training and study) were comparable. Findings indicated that there were no significant inter-group differences. Using the Cox proportional hazards model, we examined the simultaneous effect of variables on life expectancy of cases in the training and study groups, separately; the existence of metastasis, age, number of complementary treatments, presence of metastasis, and N-stage significantly affected life expectancy (P < 0.05). In the second step, survival probabilities at 6, 12, 18, 24, 36, 48, and 60 months were calculated using the Cox proportional hazards model and a NN with three nodes in the hidden layer (Figure 1). For this reason, estimates and coefficients of the Cox model and the weights of the NN model were calculated based on data of the training group, and used to derive predictions in the study group. Predictions with these two methods were compared with those of the Kaplan-Meier product limit estimator as the gold standard. Results indicated that neither prediction was significantly different from results of the Kaplan-Meier technique except probabilities predicted with the Cox method for 48 to 54 months; the 5-year survival rate of this method revealed no significant difference with standard probabilities (derived from the Kaplan-Meier product limit estimator). With the NN, the probability of survival was insignificantly higher until around 20 months compared to the standard method, and was generally lower thereafter; by the final months (around month 42), the predictions were very close to standard (Figure 1). Results were compared using the log-rank test; there was no significant difference among probabilities of survival with the three methods. Also, according to the Friedman test, there was no significant difference among the three methods in terms of the trend of changes seen in the probabilities of survival. Nevertheless, with the Kruskal-Wallis test, the 4-year (month 48) and 4.5 year (month 54) survival rates of the Cox method were significantly dissimilar from the standard methods and the NN model (P < 0.05). In the study group, mean standard errors of the probabilities of survival with the Kaplan-Meier, Cox, and NN methods were 0.03359, 0.03894, and 0.03383, respectively. The ratios of standard errors with the latter two methods to the Kaplan-Meier method were 1.1593 and 1.0071, respectively; this indicated a significant difference between Cox and Kaplan-Meier (P < 0.05), no significant differences between Cox and NN, or NN and Kaplan-Meier (P < 0.05), and enhanced accuracy for the NN (with a three node hidden layer). In the third step, we calculated probabilities of survival past 6, 12, 18, 24, 36, 48, and 60 months using three NN models with 3, 5, and 7 nodes in the hidden layer, respectively. Predictions with these three models in the study group were compared with those of the Kaplan-Meier product limit estimator as the gold standard (Figure 2). None of the three predictions were significantly different from the result of the Kaplan-Meier method, and they were almost similar by the final months (fifth year). Compared to standard, survival probabilities with these three NN models were insignificantly higher until the 20th month; generally lower thereafter, and very close near the final months of the study (around month 42). A closer examination revealed the results of 5-node hidden layer network reached the standard model around the third year (36th month), and the result of 3- and 5- node hidden layer models matched the standard model by the 42nd month, while the 7-node model gave different (although insignificant) results from the other two network models and the standard. Findings were compared using the log-rank test, and overall, there was no significant difference between probabilities of survival in the three models. Likewise, according to the comparison with the Friedman test, there was no significant difference among the 3 models in terms of the trend of changes in survival probabilities. In the study group, mean standard error of survival probabilities was 0.03359 with the Kaplan-Meier model, and 0.03383, 0.03360, and 0.03490 with the 3-, 5-, and 7-node models, respectively. The standard error ratios of the three network models to the Kaplan-Meier method were 1.0071, 1.0003, and 1.0390, respectively; this indicates lack of significant difference between any of the NNs and the standard (Kaplan-Meier), but better accuracy for the network with a 5-node hidden layer.

**Table 1.** Distribution of Independent Variables in the Training Group, the Study Group, and the Total Sample

Variable	Training Group (n = 165), No. (%)	Prediction Group (n = 165), No. (%)	Total (n = 330), No. (%)	Chi-square, Mean ± SD
**Gender**				0.06 ± 0.81
Female	52 (31.5)	50 (30.3)	102 (30.9)	
Male	113 (68.5)	115 (69.7)	228 (69.1)	
**History of smoking**				0.52 ± 0.47
No	112 (67.9)	118 (71.5)	230 (69.7)	
Yes	53 (32.1)	47 (28.5)	100 (30.3)	
**Pathology**				0.06 ± 0.44
Adenocarcinoma	143 (86.7)	138 (83.6)	281 (85.2)	
Other	22 (13.3)	27 (16.4)	49 (14.8)	
**Metastasis**				0.45 ± 0.50
Yes	93 (56.4)	99 (40.0)	192 (58.2)	
No	72 (43.6)	66 (40.0)	138 (41.8)	
**T-stage**				4.13 ± 0.25
1	10 (6.1)	3 (1.8)	13 (3.9)	
2	13 (7.9)	13 (7.9)	26 (7.9)	
3	55 (33.3)	54 (32.7)	109 (33.0)	
4	87 (52.7)	95 (57.6)	182 (55.2)	
**N-stage**				0.80 ± 0.85
0	104 (63.0)	97 (58.8)	201 (60.9)	
1	6 (3.6)	8 (4.8)	14 (4.2)	
2	44 (26.7)	47 (28.5)	91 (27.6)	
3	11 (6.7)	13 (7.9)	24 (7.3)	
**M-stage**				0.62 ± 0.43
0	149 (90.3)	153 (92.7)	302 (91.5)	
1	16 (9.7)	12 (7.3)	28 (8.5)	
**Number of complementary treatment**				5.17 ± 0.13
0	39 (23.6)	28 (17.0)	67 (20.3)	
1	42 (25.5)	34 (20.6)	76 (23.0)	
2	49 (29.7)	52 (31.5)	101 (30.6)	
3	35 (21.2)	51 (30.9)	86 (26.1)	
**Cancer site**				0.50 ± 0.78
Cardia	71 (43.0)	74 (44.8)	145 (43.9)	
Antrum	30 (18.2)	33 (20.0)	63 (19.1)	
Other	64 (38.8)	58 (35.2)	112 (37.0)	
**Final status**				1.23 ± 0.27
Alive	41 (24.8)	50 (30.3)	91 (27.6)	
Deceased	124 (75.2)	115 (69.7)	239 (72.4)	
**Age, y**	65.18 (11.32)	66.04 (10.70)	65.61 (11.01)	0.71 ± 0.48

## 5. Discussion

Predictions were extremely close to standard values when we applied the Cox proportional hazards method and a NN with a 3-node hidden layer. Survival probabilities at 6, 12, 18, 24, 36, 48, and 60 months were considered in the study group, and predictions with these two methods were compared against results with the Kaplan-Meier method as the gold standard; none of the predictions were significantly dissimilar from results with the Kaplan-Meier; exceptions were Cox probabilities for the 48th and 54th month, although the 5-year survival predicted with this method was not significantly different from standard (results from the Kaplan-Meier product limit estimator). In NN models, survival probabilities were insignificantly higher than standard until the 20th month; these probabilities were generally lower thereafter, and very close to standard near the final months of the study (around month 42) (Figure 1). The standard error ratio of Cox and NN estimates to Kaplan-Meier estimates were 1.1593 and 1.0071, respectively; this indicated noteworthy difference between standard error of estimates with the Cox and standard methods (Kaplan-Meier), and better accuracy for the NN.

Ravdin et al., who investigated on breast cancer patients' survival with NNs, agree that such models can lead to more accurate results compared to traditional methods. In their study, missing data were not considered, time was fit in the model as a predictive variable, and for each patient, and the number of survived time intervals was considered a dependent variable (9). Other independent variables were quite overlooked, and accordingly we believe our conclusions are more valid. For the first years, we observed overestimation of survival probabilities compared to standard; this has been mentioned by Ripley and Ripley as well, but they had insufficient evidence to find one model preferable to others (11). In the study by Ripley et al., where survival time until the first relapse was investigated as the dependent variable and 11 demographic, diagnostic, and treatment related variables were considered independent, variables were coded in a binary format, and the authors concluded that utilizing models based on NNs was not very beneficial (12). Results by Jones et al. indicated better accuracy for predictions of NNs compared to the Cox model; they had grouped variables binary and used three hidden layers as well (16). One of the limitations of the above two studies (Ripley and Jones) was scaling down data by converting quantitative data into binary variables; this approach was avoided in the present study. One of the debates in NN models is the number of nodes used in the hidden layer. Obviously, the upper limit is set by the number of independent variables; however, limited sample sizes and a tendency to minimize coefficient estimates had created a preference for smaller numbers of nodes. A suitable criterion to select the number of nodes would be achieving relatively accurate results with the simplest model possible. In our investigation, we used similar models which differed only in terms of the number of nodes (3, 5, and 7 nodes), and compared their results. For this purpose, survival probabilities were calculated at 6, 12, 18, 24, 36, 48, and 60 months using the three models, and we found that neither prediction was significantly different from that achieved through the Kaplan-Meier method, and they appeared to be almost similar during the final months (fifth year). From around the 42nd month, models with 3- and 5-node hidden layers almost matched the standard model, while the 7-node network generated different, (although insignificant) results compared to the other three mentioned models. Results were compared using the log-rank test, and overall, we realized no significant difference among survival probabilities with the three models, but we observed better accuracy for the NN with a 5-node hidden layer. Less accuracy for the 7-node model should be interpreted conservatively, because the smaller sample size in the training group (n = 165) may have had a certain effect. We suggest studying practical results of changing the number of nodes with larger sample sizes, which is unfortunately difficult to accomplish in survival studies, or by using bootstrap NNs. Including continuous independent variables in NN models has become simple owing to advances in computer sciences, and the application of NN based models has become promising. Predictions of survival probabilities appear to be more accurate than that achieved with the Cox proportional hazards model, especially now that many of the limitations related to performing sophisticated computations have been removed. Using these methods would be preferable due to the lack of limitations and assumptions associated with traditional models. We do not recommend adding too numerous hidden layer nodes because sample size related effects can reduce accuracy. We recommend increasing the number of nodes as far as accuracy continues to increase (decrease in mean standard error), and adding nodes should cease when a change in this trend is observed. Fitting independent variables as time-dependent ones is an issue under investigation by the authors.
